# Application of Box–Behnken Design to Optimize Phosphate Adsorption Conditions from Water onto Novel Adsorbent CS-ZL/ZrO/Fe_3_O_4_: Characterization, Equilibrium, Isotherm, Kinetic, and Desorption Studies

**DOI:** 10.3390/ijms24119754

**Published:** 2023-06-05

**Authors:** Endar Hidayat, Nur Maisarah Binti Mohamad Sarbani, Seiichiro Yonemura, Yoshiharu Mitoma, Hiroyuki Harada

**Affiliations:** 1 Graduate School of Comprehensive and Scientific Research, Prefectural University of Hiroshima, Shobara 727-0023, Japan; hidayatendar1@gmail.com (E.H.);; 2 Department of Life and Environmental Science, Faculty of Bioresources Science, Prefectural University of Hiroshima, Shobara 727-0023, Japan

**Keywords:** phosphate adsorption, zeolite, chitosan, ZrO, Fe_3_O_4_, Box–Behnken design, mechanical stability

## Abstract

Phosphate (PO_4_^3−^) is an essential nutrient in agriculture; however, it is hazardous to the environment if discharged in excess as in wastewater discharge and runoff from agriculture. Moreover, the stability of chitosan under acidic conditions remains a concern. To address these problems, CS-ZL/ZrO/Fe_3_O_4_ was synthesized using a crosslinking method as a novel adsorbent for the removal of phosphate (PO_4_^3−^) from water and to increase the stability of chitosan. The response surface methodology (RSM) with a Box–Behnken design (BBD)-based analysis of variance (ANOVA) was implemented. The ANOVA results clearly showed that the adsorption of PO_4_^3−^ onto CS-ZL/ZrO/Fe_3_O_4_ was significant (*p* ≤ 0.05), with good mechanical stability. pH, dosage, and time were the three most important factors for the removal of PO_4_^3−^. Freundlich isotherm and pseudo-second-order kinetic models generated the best equivalents for PO_4_^3−^ adsorption. The presence of coexisting ions for PO_4_^3−^ removal was also studied. The results indicated no significant effect on PO_4_^3−^ removal (*p* ≤ 0.05). After adsorption, PO_4_^3−^ was easily released by 1 M NaOH, reaching 95.77% and exhibiting a good capability over three cycles. Thus, this concept is effective for increasing the stability of chitosan and is an alternative adsorbent for the removal of PO_4_^3−^ from water.

## 1. Introduction

Phosphate (PO_4_^3−^) is a macronutrient needed for plant growth and is frequently applied as a fertilizer on agricultural lands. The increasing demands of food supply nowadays have led to the excessive application of fertilizer. However, excessive fertilizer use can cause PO_4_^3−^ to leach into waterways, leading to eutrophication and harmful algal bloom. These blooms diminish oxygen levels [1,2,3], interfere with aquatic life, and adversely affect the quality of drinking water (taste and odor) [4]. According to [5], PO_4_^3−^ decontamination must be performed efficiently while having a minimal impact on the surrounding ecosystem. Many methods have been reported to be effective in removing PO_4_^3−^ from water, including biological [6] methods, electrochemical [7,8] methods, precipitation [9], ion exchange [10], and adsorption [11,12]. Each strategy has advantages and disadvantages. Biological techniques are more economical; however, the residue of dead bacteria left behind after the process is inconvenient [13]. Electrochemical techniques are expensive but have a lower effectivity toward PO_4_^3−^ removal [14]. The precipitation process is simple and effective for chemical treatment but is inefficient for sewage sludge and waste disposal [15]. Ion exchange may also be used to remove anions by exchanging sulfates (SO_4_^2−^) for PO_4_^3−^ ions; however, this would make the solution more corrosive, and it requires a costly clean-up (Blaney et al. [16]). Adsorption is the best option and is the most widely used method for water contaminants including PO_4_^3−^ ions [17,18]. This is because the technique is environmentally safe, the operation is easy and fast, and the technology is highly efficient.

Chitosan is currently gaining popularity as a potential adsorbent for water contaminants because it contains hydroxyl (–OH) and amino (–NH_2_) functional groups, which can easily react with other materials and are environmentally friendly [19]. This material, which cannot be accessed readily from nature, is synthesized through the chemical deacetylation of chitin. However, because of its low tensile strength and dissolution under acidic conditions, the use of chitosan directly in wastewater treatment technologies is not recommended. Therefore, chitosan must be modified to increase its chemical stability and adsorption capability [20]. The selection of an appropriate modification method and modifying agent is crucial for assessing the quality and functionality of the product created during the modification process. Crosslinking is one of the most frequently used procedures to enhance the physicochemical characteristics of chitosan [21,22]. Crosslinking is the process of combining two or more molecules via covalent bonds.

Zeolites are crystalline aluminum silicate (Al_2_O_3_·2SiO_2_) minerals with a porous and highly stable structure, and they could enhance the adsorption of chitosan onto their surface, leading to the improved stability of chitosan. These materials can be obtained from natural sources, such as shrimp, or can be synthesized using various methods [23]. Several reports have proven the use of chitosan and zeolite to remove dyes [24,25], pharmaceuticals [26], nitrate [27], and humic acid [28]. On the other hand, the fabrication of chitosan–metal oxides has attracted the attention of a lot of scientists owing to their numerous beneficial characteristics, such as chemical stability, a large surface area, and favorable adsorptive characteristics [29]. Magnesium oxide (MgO) [30], titanium oxide (TiO) [31], zinc oxide (ZnO) [32,33], zirconium oxide (ZrO) [34], and copper oxide (CuO) [35] are examples of metal oxides. ZrO was selected for this study owing to its strong affinity for anions [36]. 

The separation of the adsorbents is another issue of concern since the usual separation procedures result in the loss of the adsorbents as well as possible dangers to the environment [37,38]. Magnetite (Fe_3_O_4_) is one of the most magnetic particles that can be used in the manufacture of magnetic adsorbents for water purification because of its biodegradability, thermal stability, and large surface area [39,40]. The use of the crosslinking method to combine magnetite, zeolite, ZrO, and chitosan is a viable strategy. This is because the magnetic particles allow for easy separation when subjected to an external magnetic field, while the chitosan, zeolite, and ZrO provide many adsorption sites [41]. Therefore, the amalgamation of chitosan/zeolite/ZrO, and Fe_3_O_4_ (CS-ZL/ZrO/Fe_3_O_4_) may result in the development of novel composite materials with multifunctional constituents. 

This study synthesized CS-ZL/ZrO/Fe_3_O_4_ with the target of using it as a novel adsorbent for PO_4_^3−^ removal from water. The response surface methodology (RSM) with the Box–Behnken design (BBD) optimization strategy was used to acquire insight into the effect of process factors such as pH, adsorbent dosage, temperature, and time to achieve the maximal adsorptive removal of PO_4_^3−^. This process was performed to obtain the highest PO_4_^3−^ adsorptive removal. The adsorption isotherms and kinetic models were also calculated to figure out the adsorption mechanism.

## 2. Results and Discussion

### 2.1. Characterization of CS-ZL/ZrO/Fe_3_O_4_

**Figure 1:**
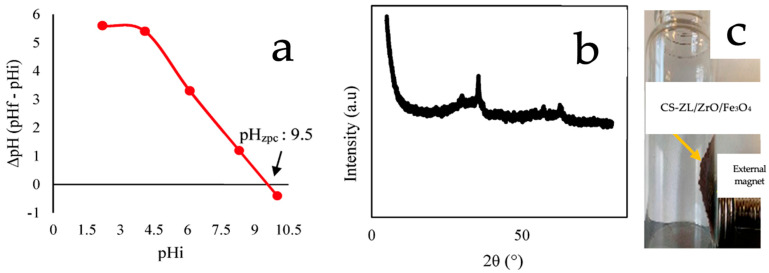
(**a**) pH_zpc_ of CS-ZL/ZrO/Fe_3_O_4_, (**b**) XRD spectra of CS-ZL/ZrO/Fe_3_O_4_, and (**c**) photograph of CS-ZL/ZrO/Fe_3_O_4_ (taken by phone).

The experimental results of BBD are listed in Table 1. It can be concluded that a pH of 2 offers the best conditions for PO_4_^3−^ removal. The pH_ZPC_ findings revealed that, at a pH of 2, the surface of CS-ZL/ZrO/Fe_3_O_4_ had a positive charge (pH < pH_zpc_) (Figure 1a). This might indicate the protonation of the -NH_2_ groups to -NH_3_^+^ groups on the surface. These attract negatively charged H_2_PO_4_^−^ ions to CS-ZL/ZrO/Fe_3_O_4_, resulting in the construction of a surface complex between PO_4_^3−^ ions and CS-ZL/ZrO/Fe_3_O_4_. This study was similar to that reported by [42,43], which used SCBC-La and leftover coal, respectively, for PO_4_^3−^ removal under acidic conditions. The other possible reaction that could occur is shown in Equation (1).

Fe_3_O_4_ + 4Zr(OH)_4_ + 6H_2_PO_4_− → FeZr(PO_4_)_3_ + 12H_2_O (1)

Table 2 summarizes the physical characteristics of these adsorbents. The results show that the BET-specific surface area was 88.1 m^2^/g, with a pore volume of 0.572 mL/g, an average diameter of 43.9 µm, and a porosity of 59%. These parameters show that the adsorbent had a substantial surface area for the adsorption of PO_4_^3−^ ions.

Figure 1b shows the XRD data used to verify the crystalline structure of the composite material. The XRD pattern shows a huge hump around 2θ = 21.22, which is a chitosan-specific peak [20]. Furthermore, the sharp peaks at 30.11, 35.49, 43.21 are mostly composed of crystalline phases, such as quartz, hematite, and alumina, which are all formed from zeolite- and zirconium-based materials. Magnetite corresponds to the peaks at 53.52, 57.08, and 62.78 [44]. Figure 1c shows a photograph of CS-ZL/ZrO/Fe_3_O_4_. It can be seen that the adsorbent is attached to the external magnet.

**Figure 2:**
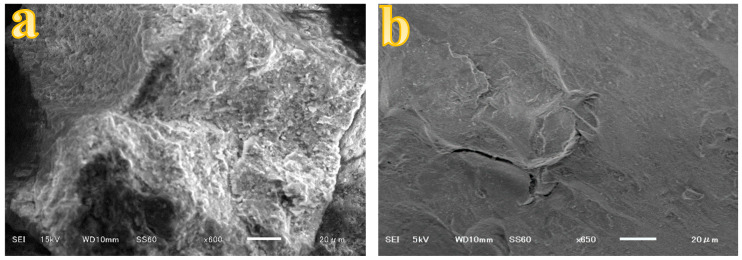
SEM images before (**a**), and after (**b**) PO_4_^3−^ adsorption.

The SEM-EDS characterization of CS-ZL/ZrO/Fe_3_O_4_ was carried out to explore the surface properties and chemical components of the material. Figure 2 and Table 3 compare the SEM images and EDS data before and after PO_4_^3−^ adsorption. Before adsorption, the surface morphology of the adsorbent was sticky, rough, and porous. The surface became smooth and compact after PO_4_^3−^ adsorption, and this indicates that PO_4_^3−^ ions were trapped on the adsorbent surface. The primary objective of the EDS data analysis was to identify the components of the adsorbent materials. The weight percentages of Zr and Fe were the highest at 50.68 and 38.92%, respectively. The N value was derived from the chitosan materials [45,46,47]. Al, Si, and Fe were derived from zeolite and magnetite, respectively. Furthermore, the presence of P after the adsorption process indicates that PO_4_^3−^ was successfully adsorbed. 

**Figure 3:**
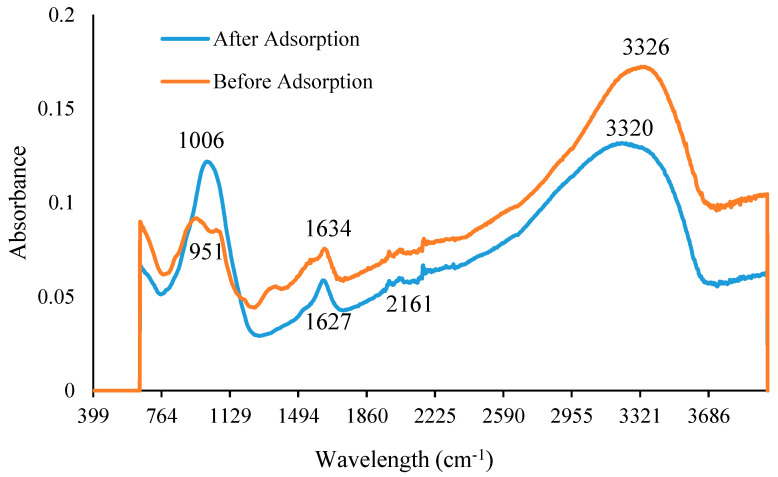
FTIR-ATR before, and after PO_4_^3−^ adsorption.

Figure 3 shows the functional groups in CS-ZL/ZrO/Fe_3_O_4_ before and after PO_4_^3−^ adsorption through an FTIR-ATR analysis. A CS-ZL/ZrO/Fe_3_O_4_ band was detected following PO_4_^3−^ adsorption from 3326 cm^−1^ to 3320 cm^−1^. This shows that PO_4_^3−^ ions interact with the stretching vibrations of hydrogen and amine in chitosan [48]. After PO_4_^3−^ adsorption, a decrease in the peak from 1634 to 1627 cm^−1^ was observed, which is associated with carboxyl groups (–COOCH_3_) [49]. An increased peak and a more curved and newer peak appeared after PO_4_^3−^ adsorption from 951 to 1006 cm^−1^ and at 2161 cm^−1^, which were assigned to Si-O-Al, Fe-O-Si, or Zr-O-Fe and CN stretching, respectively. This indicated a strong interaction with PO_4_^3−^ ions.

### 2.2. Mechanical Stability

**Figure 4:**
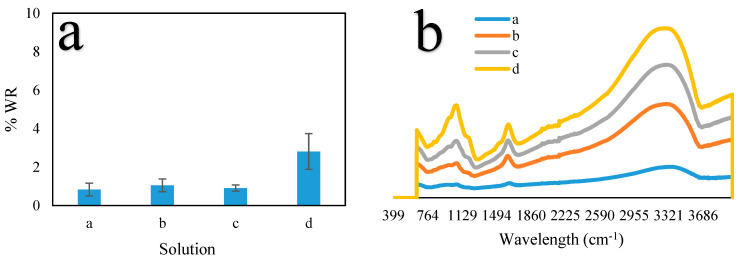
WR of CS-ZL/ZrO/Fe_3_O_4_. (**a**) Percentage WR, and (**b**) FTIR-ATR of CS-ZL/ZrO/Fe_3_O_4_ after treatment. Solution a (0.01 M HCl), b (0.1 M HCl), c (0.01 M H_2_SO_4_), and d (0.1 M H_2_SO_4_). Standard deviation (error bars).

The mechanical stability of the CS-ZL/ZrO/Fe_3_O_4_ composite was determined through the percentage of the initial mass that was preserved after drying. Figure 4a shows that increasing the concentration of the solution led to a higher WR%. Compared to the HCl-containing solution, the H_2_SO_4_-containing solution exhibited a higher WR%. Consequently, the crystalline structure of CS-ZL/ZrO/Fe_3_O_4_ was deformed, indicating that H_2_SO_4_ had significant contact with the chitosan group. Figure 4b shows the IR spectra after treatment. The positions of the peaks were consistent for all the samples. According to [50], the broad band visible at 3176–3345 cm^−1^ is assigned to the -NH_2_ groups changing to –NH_3_^+^ groups. The peaks between 1611 and 1630 cm^−1^, which were ascribed to the carboxyl (–COOCH_3_) and –NH_2_ groups, were generated through H^+^ generation by HCl and H_2_SO_4_. The peak shifted to 1068 cm^−1^, and expansion occurred when treated with 0.1 M H_2_SO_4_. SO_4_^2−^ ions have been shown to be associated with Si, Al, Fe, and Zr [51]. According to these results, the physical and chemical characteristics of the CS-ZL/ZrO/Fe_3_O_4_ composites did not change significantly. 

### 2.3. ANOVA and Equations for Fitting Empirical Models

Table 4 shows the results of the statistical analysis, using the ANOVA test to evaluate the relationship between the input effective variables (A, B, C, and D) and their responses (Y). Table 4 shows that the F-value of the quadratic model was 16.68 and that the *p*-value was < 0.0001, indicating that this model was significant. Models A, B, D, A^2^, C^2^, D^2^, A × B, A × D, and C × D, marked with an asterisk (*), were found to be significant parameters of the model. The statistical variables obtained from the ANOVA test (Equation (2)) provide a full quadratic regression model for PO_4_^3−^ removal (%).

PO_4_^3^− removal (%) = 99.2 − 1.72 A + 63 B − 1.478 C − 0.472 D + 0.2333 A^2^ + 840 B^2^ + 0.01575 C^2^ + 0.00661 D^2^ − 17.23 A*B − 0.0123A*C − 0.02107 A*D − 1.02 B*C + 2.098 B*D + 0.00343 C*D (2)

The coefficients in the equation with positive and negative signs describe the additive and multiplicative effects of the variables on the response. The “Lack of Fit” was determined by comparing the residual error to the pure error in close proximity to the repeatedly designed points. F = 3.05 and *p* = 0.272 for the “Lack of Fit” revealed that it was not significant for the model (*p* < 0.05). It can be assumed that the model was adequately fitted and that there was no lack of fit. 

**Figure 5:**
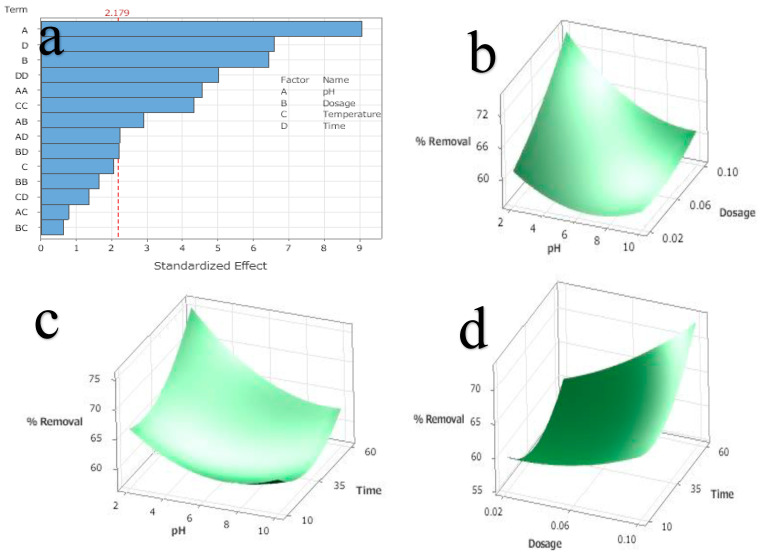
(**a**) Pareto chart for the standardized effect of various factors on PO_4_^3−^ removal by adsorbent, (**b**) pH and dosage of adsorbent response surface’s effect on PO_4_^3−^ removal (%), (**c**) pH and time response surface’s effect on PO_4_^3−^ removal (%), and (**d**) pH and dosage of adsorbent and time response surface’s effect on PO_4_^3−^ removal (%).

The R^2^ value of the calculated second-order response model was 95.11, which is also known as the coefficient of determination. Consequently, it can be applied to reliably calculate the response at any given parameter level regardless of their values. In addition, a regression model was used to calculate the standardized influence of the independent factors on PO_4_^3−^ removal. A response surface plot was generated to investigate the influence of two components at initial the PO_4_^3−^ concentration of 20 mg/L (Figure 5). This plot was used to determine the standardized effects of all the independent variables. A surface plot is an easier way to display the response behavior that occurs when two parameters are simultaneously altered at the same time. It is more beneficial to select the quantity of various ingredients to obtain the desired response. Figure 5a displays a Pareto chart that compares the relative magnitude and the corresponding main, square, and interaction effects of the selected variables. The square effects of all four factors were found to be very significant (*p* ≤ 0.05) in addition to the main effects of the factors, which were also found to be highly significant (*p* ≤ 0.05). The ANOVA results reported in Table 4 led to the same conclusions. PO_4_^3−^ removal continuously increased in response to the pH, adsorbent dosage, and time. Figure 5b,c show that pH is a key factor in the removal of PO_4_^3−^, and Figure 5d shows that increasing the contact time increases the percentage removal. 

### 2.4. Initial Concentration and Isotherm Studies

**Figure 6:**
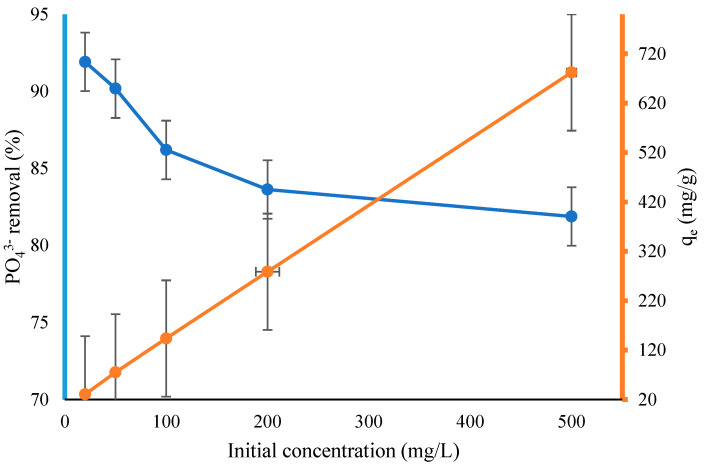
Effect of initial concentration on PO_4_^3−^ removal onto CS-ZL/ZrO/Fe_3_O_4_. Standard deviation (error bars).

The effects of the initial PO_4_^3−^ concentrations ranging from 20 to 500 mg/L, 0.06 g of adsorbent (CS-ZL/ZrO/Fe_3_O_4_), and pH (2.0) were investigated. Figure 6 shows that the PO_4_^3−^ adsorption capacity rose from 30.64 to 682.31 mg/g; however, the percentage of PO_4_^3−^ removal decreased from 91.91% to 81.88%. The adsorption capacity increased with the concentration because the total number of molecules increased. Consequently, the mass transfer resistance of adsorbate decreased. As a result, the percentage of removal decreased [52]. 

Adsorption isotherms are necessary to assess the capabilities of an adsorbent and the interactions between an adsorbate (a solution containing PO_4_^3−^ ions) and an adsorbent (CS-ZL/ZrO/Fe_3_O_4_). The acquired isotherm parameters can be used to conduct an accurate analysis while constructing an effective adsorption system. Both the equilibrium concentration and the adsorption capacity were estimated. The Langmuir model describes the monolayer adsorption processes at the designated homogenous surfaces on the adsorbent (Equation (3)). The essential property of the Langmuir model can be described as a dimensionless constant also known as the separation factor (R_L_), which is shown in Equation (4). By contrast, the Freundlich model is based on heterogeneous surfaces and multilayer sorption (Equation (5)). This is a linear equation, which is shown as follows:

(3) $$C_{e}{/q}_{e}=\left(\frac{C_{e}}{q_{\max}}\right)+1/\left(K_{1}q_{\max}\right)$$

(4) $$R_{L}=(\frac{1}{{1+\mathrm{bC}}_{o}})\;$$

(5) $${\mathrm{Ln}\;q=\mathrm{lnK}}_{f}+\frac{1}{n}x\;{\mathrm{lnC}}_{e}$$

q_e_ (mg/g) is the adsorption capacity; K_1_ (L/mg) is the equilibrium constant of adsorption; q_max_ (mg/g) is the maximal adsorption capacity; C_e_ (mg/L) is the equilibrium concentration; C_o_ (mg/L) is the initial concentration; R_L_ is the separation factor; 0 < R_L_ is favorable; R_L_ > 1 is unfavorable; R_L_ = 1 is linear; and K_f_ (mg/g) and 1/n are the adsorption capacity and the intensity of adsorption, respectively. 

**Figure 7:**
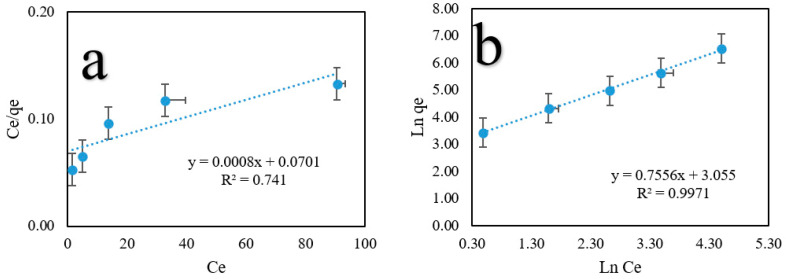
Linear curves of PO_4_^3−^ adsorption isotherm models. (**a**) Langmuir, and (**b**) Freundlich models. Standard deviation (error bars).

Figure 7 shows the isotherm model curves, and Table 5 shows the fitting results corresponding to these curves. The Freundlich model’s linear correlation coefficient (R^2^) was 0.9970, indicating that it provided the best fit compared to the other models. More importantly, the Langmuir and Freundlich parameters, known as R_L_ and 1/n, indicate that the PO_4_^3−^ ion has a type of < 1. According to these data, the PO_4_^3−^ adsorption method is favorable.

### 2.5. Adsorption Kinetic Studies

**Figure 8:**
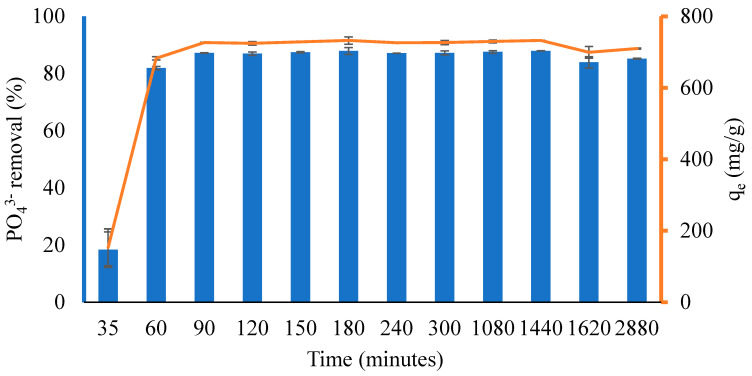
The effect of contact time on PO_4_^3−^ removal onto CS-ZL/ZrO/Fe_3_O_4_. Standard deviation (error bars).

This study investigated the influence of the contact time (35–2880 min) on PO_4_^3−^ adsorption at 30 °C. Figure 8 shows that the percentage of PO_4_^3−^ removal and the capacity for adsorption increased rapidly from 35 to 60 min and then gradually increased up to 90 min. This is because the adsorbent includes carboxyl, amine, hydrogen, and magnetite groups, all of which cause the adsorbent surface to become active and trap PO_4_^3−^ ions. Subsequently, the adsorption capacity decreased and increased, resulting in fast/slow PO_4_^3−^ adsorption, and it finally reached equilibrium at 1440 min, with an adsorption capacity and percent removal of 732.56 mg/g and 87.91%, respectively.

Adsorption kinetic studies are important because they deliver information on the adsorption mechanism, which is necessary to assess the effectiveness of the process [53]. Two kinetic models were used in this study: pseudo-first-order (PFO) (Equation (6)) and pseudo-second-order (PSO) (Equation (7)) models were investigated. The linear form can be obtained by calculating the following equation.

(6) $$\mathrm{Log}\left(q_{e}-{\;q}_{t}\right)=\log q_{e}-K_{1}t$$

(7) $$t/q_{t}=1/\left(K_{2}{q_{e}}^{2}\right)+t/q_{e}$$

where k_1_ (min^−1^) is the rate constant of the PFO model, t (min) is the time, and a linear plot of log t against log (q_e_ − q_t_) and t against t/q_t_ was used to determine K_1_ and K_2_ from the slope of the linear plots, respectively.

**Figure 9:**
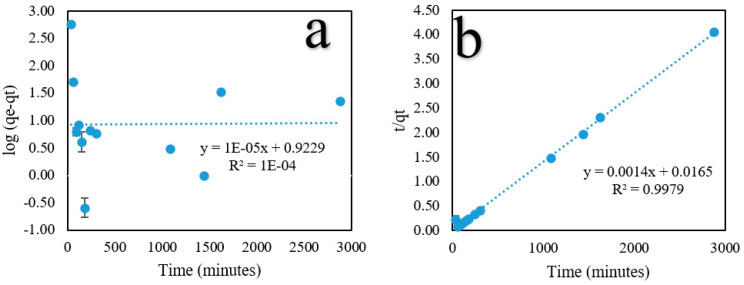
Linear curves of PO_4_^3−^ adsorption kinetic studies. (**a**) Pseudo-first-order (PFO) and (**b**) pseudo-second-order (PSO) models. Standard deviation (error bars).

Figure 9 shows the fitting curves for the kinetic models, and Table 6 lists the fitting results corresponding to those curves. The findings confirm that the PSO model provided better results than the PFO model in terms of the linear correlation coefficient R^2^ value (0.9979). These findings imply that chemical processes control the adsorption rate.

### 2.6. Effect of Anions and Cations on PO_4_^3−^ Removal onto CS-ZL/ZrO/Fe_3_O_4_

**Figure 10:**
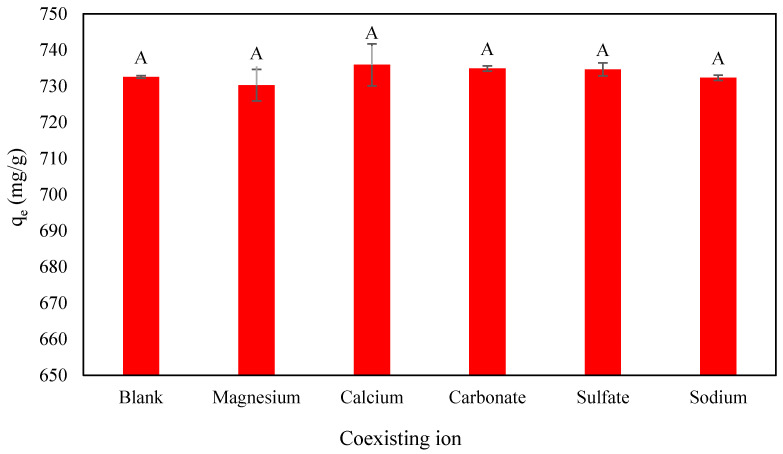
The effect of coexisting ions on PO_4_^3−^ removal onto CS-ZL/ZrO/Fe_3_O_4_. Standard deviation (error bars). A: no significant effect (*p* ≤ 0.05).

Wastewater contains various substances, including anions and cations, which can affect the adsorption process [54]; it is essential to investigate the effect of ionic strength in an aqueous solution. This is because wastewater is made up of numerous components that might be found together. Figure 10 depicts the effect of different anions and cations on the PO_4_^3−^ adsorption capacity of CS-ZL/ZrO/Fe_3_O_4_. The experimental data indicate that there was no significant influence on PO_4_^3−^ removal. It revealed that the fabrication of CS-ZL/ZrO/Fe_3_O_4_ was effective in eliminating PO_4_^3−^ from water.

### 2.7. Desorption Studies

**Figure 11:**
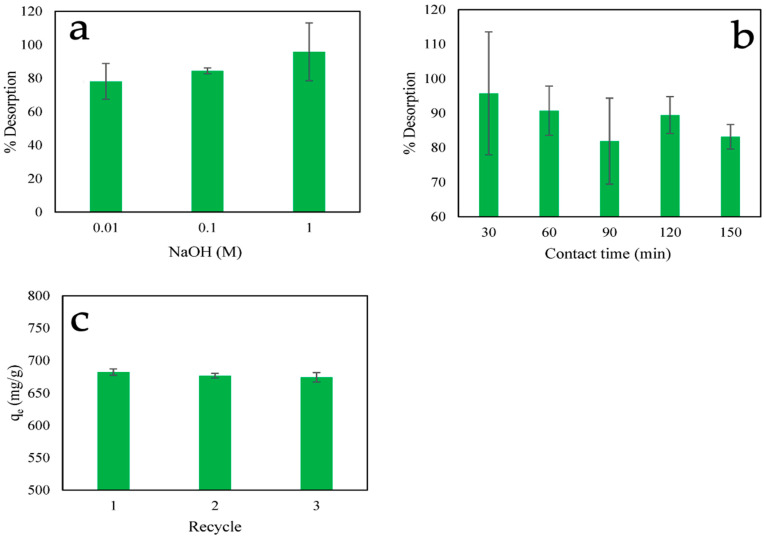
The percentage of desorption. (**a**) Different NaOH concentrations and (**b**) different contact times using 1 M NaOH, and (**c**) in recycle studies on PO_4_^3−^ adsorption capacity. Standard deviation (error bars).

Figure 11a shows the desorption percentage of PO_4_^3−^ at different NaOH concentrations from 0.01 M to 1 M for 30 min at 30 °C. The results indicate that increasing the concentration increased the desorption percentage to 95.77%. Then, subsequent experiment at different contact times, from 30 to 150 min, using 1 M NaOH (Figure 11b). The desorption percentage increased and then decreased up to 150 min, which is similar to the results of the adsorption studies. The highest desorption percentage was observed after 30 min. The desorption mechanism may cause the hydroxide ions (OH-) in the sodium hydroxide solution to react with the CS-ZL/ZrO/Fe_3_O_4_-P surface and replace the PO_4_^3−^ groups, resulting in the release of PO_4_^3−^ into the liquid solution (Equation (8)). The reusability studies of PO_4_^3−^ adsorption onto CS-ZL/ZrO/Fe_3_O_4_ showed good performance for three cycles (Figure 11c).

H_2_PO_4_^−^ + OH^−^ → HPO_4_^2−^ + H_2_O (8)

### 2.8. Adsorption Performance Comparison

Table 7 compares the equilibrium and maximum adsorption capacity of CS-ZL/ZrO/Fe_3_O_4_ with those of various adsorbents. It can be seen that the pH is one of the main factors for PO_4_^3−^ removal onto the adsorbent, and the surface charge can become either positive or negative over a wide pH range, which influences the interaction between the adsorbent and PO_4_^3−^ ions. It is clear that the CS-ZL/ZrO/Fe_3_O_4_ adsorbent has a higher capacity than the other adsorbents. It is feasible to conclude that these adsorbents are viable alternatives for removing PO_4_^3−^ from water.

## 3. Materials and Methods

### 3.1. Materials

Chitosan (CH) (C_6_H_11_NO_4_) with molecular weight of 100,000–300,000 Da was bought from Acros Organics, Belgium. Zeolite (ZL) (Al_2_O_3_·2SiO_2_) was obtained from Tosoh Co. Ltd., Japan. Sodium hydroxide (NaOH), acetic acid (CH_3_COOH), disodium hydrogen phosphate (Na_2_HPO_4_), ferric chloride (FeCl_3_), ferrous sulfate (Fe_2_SO_4_), ammonium molybdate ((NH_4_)_6_Mo_7_O_24_·4H_2_O)), antimony potassium tartrate (K_2_Sb_2_(C_4_H_2_O_6_)_2_), ascorbic acid (C_6_H_8_O_6_), hydrochloric acid (HCl), and sulfuric acid (H_2_SO_4_) were bought from Kanto Chemical Co., Inc., Tokyo, Japan. ZrClO was purchased from Fujifilm Wako Chemical, Tokyo, Japan. 

### 3.2. Synthesis of CS-ZL/ZrO/Fe_3_O_4_

CS-ZL/ZrO/Fe_3_O_4_ was synthesized through crosslinking method; chitosan (1 g) was dissolved in 100 mL of acetic acid (1%), and the resulting viscous solution was maintained at ambient temperature (25–30 °C) with magnetic stirring for 24 h (Equation (9)). Subsequently, 25 mL of the resulting chitosan solution was mixed with 0.5 g of zeolite and 20 mL of 1 M FeCl_3_ + 0.5 M Fe_2_SO_4_ + 0.5 M ZrClO. The mixture solution was then heated to 60 °C and was stirred for 1 h. The pH of the solution was adjusted to 10 using 3 M NaOH over 24 h with magnetic stirring at ambient temperature (25–30 °C), and the solution was filtered and washed multiple times with acetone and distilled water (DW) to remove any remaining NaOH. Subsequently, the materials were dried for 48 h in an oven at 60 °C (Equation (13)). The adsorbents are referred to as CS-ZL/ZrO/Fe_3_O_4_.

(CH_3_COOH)n + (C_6_H_11_NO_4_)m → (CH_3_COO^−^)n(C_6_H_11_NO_4_H^+^)m (9)

(C_6_H_11_NO_4_H^+^)n + Al_2_O_3_·2SiO_2_ → (C_6_H_11_NO_4_ − Al_2_O_3_·2SiO_2_)n + 2H_2_O (10)

5FeCl_3_ + 15Fe_2_(SO_4_)_3_ + 12NaOH → 5Fe_3_O_4_ + 15Na_2_SO_4_ + 6H_2_O + 36NaCl (11)

FeCl_3_ + 3Fe_2_(SO_4_)_3_ + ZrClO + 14NaOH → 5Fe_3_O_4_ + Zr(OH)_4_ + 2Na_2_SO_4_ + 6NaCl + 7H_2_O (12)

2(CH_3_COO^−^)n(C_6_H_11_NO_4_H^+^)m + 3Al_2_O_3_·2SiO_2_ + 3FeCl_3_ + Fe_2_SO_4_ + ZrClO_4_ + 14NaOH → [3Al_2_O_3_·2SiO_2_ −(C_6_H_11_NO_4_)]2m/3·Fe_3_O_4_·xH_2_O + 3Fe(OH)_3_ + 2Zr(OH)_4_ + 6NaCl + (2n + 2m)CH_3_COONa + (2n + m)H_2_O (13)

Following this reaction, the negatively charged surface of the zeolite (Al_2_O_3_.2SiO_2_) may interact with the positively charged chitosan to produce chitosan–aluminosilicate complex. Electrostatic interactions between Fe^3+^ and Zr^4+^ ions and chitosan are another mechanism by which chitosan combines with metal ions to form chitosan–metal complexes. Fe(OH)_3_ and Fe_3_O_4_ are formed when Fe^2+^ and Fe^3+^ ions react with hydroxide ions (OH^−^) from NaOH.

### 3.3. The Design of the Experiment

Experiments were conducted using response surface methodology (RSM) in combination with Box–Behnken design (BBD), and statistical analysis was performed using Minitab 21.3.1 software. (A) The pH (2–10), (B) dosage (0.02–0.1 g), (C) temperature (30–60 °C), and (D) contact time (10–60 min) were the independent variables examined in the BBD, with three levels and four parameters (Table 8). In total, 27 different sets of experiments were performed to determine the optimal conditions for PO_4_^3−^ removal. The data obtained were assessed using an equation for a quadratic polynomial response surface, which was calculated using Equation (14), to identify the relationships between independent variables and response.

(14) $${Y=E}_{0}{+E}_{1}{A+E}_{2}{B+E}_{3}{C+E}_{4}{D+E}_{11}A^{2}{+E}_{22}B^{2\;}{+E}_{33}C^{2}{+E}_{12}{\mathrm{AB}+E}_{13}{\mathrm{AC}+E}_{23}\mathrm{BC}+\varepsilon$$

The coefficients of the polynomial model are represented as follows: E0 is constant expression, E1–E3 are linear effects, E11–E33 are second-order effects, E12–E23 are interactive effects, and ε is error term. An analysis of variance (ANOVA) was performed to calculate the F- and *p*-values of the model to measure its statistical significance and appropriateness. The statistical significance of the model is shown through the model’s F-value and *p*-value, and a lack-of-fit study of the proposed model was executed using Minitab 21.3.1 software. In addition, a 3D response surface plot and Pareto chart of standardized effects were developed to figure out the cooperative quantitative impact of the independent variables on the response and overall value of the model [63].

### 3.4. Batch Adsorption Study and Response Determination (PO_4_^3−^ Removal %)

To evaluate the efficiency of PO_4_^3−^ removal, batch adsorption approach was used in this study. In total, 100 mL of PO_4_^3−^ (20 mg/L) was placed in a 300 mL conical flask. After the adsorption procedure was completed, external magnetite was placed in the conical flask to separate the adsorbent and adsorbate. PO_4_^3−^ removal was calculated using Equation (15).

(15) $${{\mathrm{PO}}_{4}}^{3-}\;\mathrm{removal}\;\%=\frac{C_{o}-C_{e}}{C_{o}}\;\times \;100$$

where C_o_ and C_e_ are the initial and equilibrium PO_4_^3−^ concentrations (mg/L), respectively.

The data from run 17 of the BBD were used for subsequent experiments (isotherm and kinetic models). However, 30 min was not used because the results were far from equilibrium. The amount of PO_4_^3−^ adsorbed was determined using Equation (16).

(16) $$q_{e}=\frac{C_{o}-C_{e}}{W}\;\times \;V$$

where q_e_ (mg/g) is the adsorption capacity, W (g) is the amount of CS-ZL/ZrO/Fe_3_O_4_, and V (L) is the volume of adsorbate (PO_4_^3−^ solution). 

### 3.5. Adsorption Isotherm Studies

The isotherm model was studied with PO_4_^3−^ solutions ranging from 20 mg/L to 500 mg/L with pH of 2. These examinations were performed for 60 min at 30 °C, and adsorbent dosage of 0.06 g was placed in the flask. In this work, Langmuir and Freundlich models were used to assess PO_4_^3−^ adsorption onto CS-ZL/ZrO/Fe_3_O_4_ [64].

### 3.6. Adsorption Kinetic Studies

Pseudo-first-order (PFO) and pseudo-second-order (PSO) models were used to investigate the model of adsorption kinetics. The following parameters were used in the experiment: an adsorption temperature of 30 °C, an initial PO_4_^3−^ concentration of 500 mg/L at pH of 2, an adsorbent dosage of 0.06 g, and contact time ranging from 35 to 2880 min. 

### 3.7. Influence of Coexisting Ionic Strength

The experiment was conducted under optimum conditions with a dosage of 0.06 g, an initial PO_4_^3−^ concentration of 500 mg/L, and a contact time of 1440 min at 30 °C. The coexisting ion was prepared with cationic and anionic ions at a concentration of 20 mg/L (Mg^2+^, Ca^2+^, CO_3_^2−^, SO_4_^−^, and Na^+^). 

### 3.8. Desorption and Reusability Studies

In most practical applications, it is essential to employ adsorbents with high level of reusability. NaOH was chosen as desorbing agent to release PO_4_^3−^ ion from CS-ZL/ZrO/Fe_3_O_4_. Firstly, 0.06 g of CS-ZL/ZrO/Fe_3_O_4_ was loaded with 500 mg/L of PO_4_^3−^ ion at pH of 2.0, which was called CS-ZL/ZrO/Fe_3_O_4_-P. Then, 0.01 g of CS-ZL/ZrO/Fe_3_O_4_-P was dispersed in 60 mL of NaOH at 30 °C. The desorption capacity and desorption percentage are shown in Equations (17) and (18), respectively. Reusability was assessed using the same treatment as described above.

(17) $$q_{\mathrm{des}}=\frac{C}{W}\;\times \;V$$

(18) $$\%\;\mathrm{Desorption}=\frac{q_{\mathrm{des}}}{q_{e}}\;\times \;100$$

where q_des_ (mg/g) is the desorption capacity; C (mg/L) is the PO_4_^3−^ concentration of desorption; % Desorption (%) is the percentage desorption; and W, V, and q_e_ are the same as above.

### 3.9. PO_4_^3−^ Measurements

PO_4_^3−^ ions were measured using the molybdate blue method. A total of 12 g of (NH_4_)_6_Mo_7_O_24_·4H_2_O was mixed with 100 mL of DW. K_2_Sb_2_(C_4_H_2_O_6_)_2_ (0.277 g) was added followed by 140 mL of 18 M H_2_SO_4_. Afterward, it was adjusted to 1 L with distilled water (solution A). A total of 1.06 g of C_6_H_8_O_6_ was added to and mixed with 100 mL of solution A, 25 mL of 4 N H_2_SO_4_ was added, and the solution was adjusted to 1 L with DW (solution B). Note: This solution must be prepared in every experiment. The procedure for the mixed solution was as follows: 2 mL of liquid sample/standard was mixed with 10 mL of solution B. Afterwards, we waited for 30 min and then analyzed the solution using a UV-Vis spectrophotometer (Jasco V-530) at a wavelength of 693 nm. A standard curve for PO_4_^3−^ was constructed using Na_2_HPO_4_.

### 3.10. Mechanical Stability

The mechanical stability of the CS-ZL/ZrO/Fe_3_O_4_ composite was evaluated based on the responses of the samples to a water bath shaker at 80 °C. For one hour, dried CS-ZL/ZrO/Fe_3_O_4_ was soaked in HCl and H_2_SO_4_ concentrations ranging from 0.01 to 0.1 M. Following that, the sample was dried in an oven at 60 °C for twenty-four hours. The calculation of the dry weight retention (WR) was performed using Equation (19).

(19) $$\mathrm{WR}\;(\%)=\frac{w_{i}}{w_{a}}\;\times \;100$$

where w_i_ and w_a_ are the dry weights of CS-ZL/ZrO/Fe_3_O_4_ before and after treatment, respectively. 

### 3.11. Characterization of CS-ZL/ZrO/Fe_3_O_4_

The crystalline structure of CS-ZL/ZrO/Fe_3_O_4_ was analyzed using a powder X-ray diffractometer (XRD) equipped with Cu/Kα radiation (Hypix-3000). Fourier transform infrared spectra (FTIR) of CS-ZL/ZrO/Fe_3_O_4_ were measured before and after PO_4_^3−^ adsorption using a Thermo Scientific Nicolet iS10 instrument (Thermo Fisher Scientific Inc., Waltham, MA, USA). The ATR-FTIR approach was used to analyze samples with a resolution of 4 cm^−1^ throughout the wavenumber spectrum spanning 400–4000 cm^−1^. To determine the specific surface area (SSA), the BET approach was combined with a surface area analyzer (MicroActive AutoPore V 9600 2.03.00, Micromeritics, Norcross, GA, USA). SEM-EDS (JIED-2300, Shimadzu, Kyoto, Japan) was used to examine the SEM images and the elemental distributions of CS-ZL/ZrO/Fe_3_O_4_. The initial (pHi) and final (pHf) pH values of the solutions were measured to determine the surface charge over a range of pH values (pH_zpc_). The pHi was adjusted from 2.0 to 10.0 in 0.01 M NaCl solution. Following that, 0.1 g of CS-ZL/ZrO/Fe_3_O_4_ was added and stirred for 24 h at 30 °C, and pHf was measured. A plot of ΔpH = pHf − pHi vs. pHi was used to determine pH_pzc_, which corresponds to the neutral surface charge. 

### 3.12. Data Analysis

All results were noted and edited using Microsoft Excel. The effects of coexisting ions on PO_4_^3−^ removal were examined using a completely randomized design (CRD). Data were analyzed using ANOVA with Tukey’s test (*p* ≤ 0.05) using Minitab 21.3.1.

## 4. Conclusions

In this study, a novel adsorbent, CS-ZL/ZrO/Fe_3_O_4_, was prepared from chitosan (CS), zeolite (ZL), ZrO, and magnetite (Fe_3_O_4_) via a crosslinking approach. The Box–Behnken design (BBD) and the response surface methodology (RSM), with their corresponding four separate factors (pH, dosage, temperature, and time), were used to develop the best experimental conditions for PO_4_^3−^ removal. Weight retention (WR) was measured in a batch reactor under acidic conditions (HCl and H_2_SO_4_) at 80 °C for 1 h to determine the mechanical stability. The results indicate that CS-ZL/ZrO/Fe_3_O_4_ was stable and did not change in the functional group peak area after treatment. The best conditions were at a pH of 2.0, with an adsorption capacity and percentage removal of 732.56 mg/g and 87.91%, respectively. The Freundlich isotherm and pseudo-second-order (PSO) kinetic models were fitted to PO_4_^3−^ removal, indicating heterogeneous and chemical sorption. In addition, the results suggest that PO_4_^3−^ adsorption occurred via the electrostatic interactions between the positive charge of CS-ZL/ZrO/Fe_3_O_4_ and the negative charge of H_2_PO^4−^ as well as ion exchange and hydrogen bonding. The presence of coexisting ions (Mg^2+^, Ca^2+^, CO_3_^2−^, SO_4_^2−^, and Na^+^) had no effect on the removal of PO_4_^3−^ (*p* ≤ 0.05). The desorption studies revealed that 1 M NaOH was better at releasing PO_4_^3−^, reaching 95.77% after 30 min of treatment at 30 °C. The reusability of CS-ZL/ZrO/Fe_3_O_4_ showed good performance over three cycles. These findings imply that CS-ZL/ZrO/Fe_3_O_4_ is the best way to improve the stability of chitosan under acidic conditions, and it is a good adsorbent for removing PO_4_^3−^ and other potential water pollutants from water.

## Figures and Tables

**Table 1:** Experimental data results from 4 factors of BBD for PO_4_^3−^ removal onto CS-ZL/ZrO/Fe_3_O_4_.

Run	pH	Dosage	Temperature	Time	% Removal
1	2	0.02	45	35	58.95
2	10	0.02	45	35	51.75
3	2	0.10	45	35	72.77
4	10	0.10	45	35	54.54
5	7	0.06	30	10	61.83
6	7	0.06	60	10	56.68
7	7	0.06	30	60	64.95
8	7	0.06	60	60	64.94
9	2	0.06	45	10	59.09
10	10	0.06	45	10	54.34
11	2	0.06	45	60	72.75
12	10	0.06	45	60	59.57
13	7	0.02	30	35	56.02
14	7	0.10	30	35	61.46
15	7	0.02	60	35	56.69
16	7	0.10	60	35	59.69
17	2	0.06	30	35	66.84
18	10	0.06	30	35	60.16
19	2	0.06	60	35	64.70
20	10	0.06	60	35	55.08
21	7	0.02	45	10	54.40
22	7	0.10	45	10	58.87
23	7	0.02	45	60	56.72
24	7	0.10	45	60	69.58
25	7	0.06	45	35	53.48
26	7	0.06	45	35	55.47
27	7	0.06	45	35	53.47

**Table 2:** Physical properties of the adsorbent.

Specific Surface Area	Value
BET-specific surface area (m^2^/g)	88.1
Pore volume (mL/g)	0.572
Average diameter (µm)	43.9
Porosity (%)	59

**Table 3:** EDS data before and after PO_4_^3−^ adsorption.

Element	Weight %		Atomic %	
	Before	After	Before	After
N	3.27	2.06	13.43	7.66
Al	0.78	0.93	1.65	1.79
Si	6.35	1.70	12.98	3.15
Fe	38.92	56.39	40.03	52.69
Zr	50.68	27.76	31.91	15.88
P		11.17		18.82

**Table 4:** ANOVA results for PO_4_^3−^ removal.

Source	DF	Sum of Squares	Mean of Squares	F-Value	*p*-Value	Remarks
Model	14	839.751	59.982	16.68	<0.0001 *	Significant
A	1	296.610	296.610	51.8093	<0.0001 *	
B	1	149.672	149.672	12.5582	<0.0001 *	
C	1	15.143	15.143	1.2835	0.063	
D	1	156.241	156.241	0.6342	<0.0001 *	
A^2^	1	74.285	74.285	12.8151	0.001 *	
B^2^	1	9.642	9.642	1.4722	0.127	
C^2^	1	67.008	67.008	7.1279	0.001 *	
D^2^	1	90.952	90.952	1.9374	<0.0001 *	
A × B	1	30.415	30.415	4.2734	0.013 *	
A × C	1	2.161	2.161	0.3036	0.453	
A × D	1	17.766	17.766	8.2118	0.046 *	
B × C	1	1.488	1.488	0.2091	0.532	
B × D	1	17.598	17.598	0.0540	0.047 *	
C × D	1	6.605	6.605	1.84	0.200	
Error	12	43.157	3.596			
Lack-of-Fit	10	40.504	4.050	3.05	0.272	
Pure Error	2	2.653	1.3267			
Total	26	882.908				
R^2^		95.11				
R^2^ adj		89.41				

* Significant.

**Table 5:** Isotherm model parameters for PO_4_^3−^ removal onto CS-ZL/ZrO/Fe_3_O_4_.

Isotherms	Parameters	Value
Langmuir	q_max_	1259.79
	K_L_	14.27
	R^2^	0.7409
	R_L_	0.0007
Freundlich	K_f_	1135.07
	1/n	0.7555
	R^2^	0.9970

**Table 6:** Kinetic model parameters for PO_4_^3−^ removal onto CS-ZL/ZrO/Fe_3_O_4_.

Kinetics	Parameters	Value
PFO	q_e_	2.5165
	K_1_	1.42857 × 10^−6^
	R^2^	1.00 × 10^−4^
PSO	q_e_	510,204.1
	K_2_	0.000119
	R^2^	0.9979

**Table 7:** List comparing PO_4_^3−^ adsorption amounts.

Adsorbent	pH	q_e_ (mg/g)	References
Magnetic iron oxide nanoparticles	11	5.03	[1]
Fe-HNT	4	5.46	[18]
Halloysite	4	3.56	[18]
20MMSB	4	121.25	[55]
Amine-functionalized nano magnetic Fe_3_O_4_ polymer	3.0	102.04	[56]
MFB-MCs	3.0	487.99	[57]
Fe_3_O_4_@SiO_2_ core/shell magnetic nanoparticles	-	27.8	[58]
AgNPs-TAC	3	13.62	[59]
Ce_0.8_Zr_0.2_O_2_	6.2	112.23	[60]
Zr/Al-Mt	5.0	17.2	[61]
PZC 7.3	11	2.41	[62]
Zeolite	11	0.69	[62]
Biochar	11	3.60	[62]
CS-ZL/ZrO/Fe_3_O_4_	2	732.56	Present study

**Table 8:** Variables and levels.

Symbol	Factor	Level 1 (− 1)	Level 2 (0)	Level 3 (+ 1)
A	pH	2	7	10
B	Dosage (g)	0.02	0.06	0.10
C	Temperature (°C)	30	45	60
D	Time (min)	10	35	60

## Data Availability

Not applicable.
